# Vitamin D Deficiency Is Associated with Endoscopic Severity in Patients with Crohn's Disease

**DOI:** 10.1155/2017/4869718

**Published:** 2017-11-16

**Authors:** Lingna Ye, Ziwen Lin, Jing Liu, Qian Cao

**Affiliations:** ^1^Department of Gastroenterology, Xiasha Branch of Sir Run Run Shaw Hospital, School of Medicine, Zhejiang University, Hangzhou, China; ^2^Inflammatory Bowel Disease Center, Sir Run Run Shaw Hospital, School of Medicine, Zhejiang University, Hangzhou, China

## Abstract

**Background and Aims:**

Vitamin D deficiency is common in patients with Crohn's disease and is associated with disease activity. Relationship between vitamin D and endoscopic disease activity is unknown. The aim of the study is to determine the association between vitamin D status and endoscopic disease activity in CD patients.

**Methods:**

Consecutive hospitalized CD patients from 2014 to 2016 who received vitamin D assessment and colonoscopy were retrospectively evaluated. Clinical disease activity was assessed by Crohn's disease activity index and C-reactive protein. Endoscopic activity was calculated using simple endoscopic score for Crohn's disease.

**Results:**

Median serum 25OHD level of 131 patients was lower than healthy controls [21.1 nmol/L (11.8–32.3) versus 49.9 nmol/L (44.9–57.4), *P* = 0.007]. 125 (95%) patients had vitamin D deficiency and the rest (5%) had vitamin D insufficiency. Serum 25OHD was inversely correlated with CRP (*r* = −0.308, *P* < 0.001), CDAI (*r* = −0.582, *P* < 0.001), SES-CD (*r* = −0.294, *P* = 0.001), and endoscopic severity stratified by SES-CD (*P* = 0.001).

**Conclusion:**

Vitamin D deficiency was prevalent among hospitalized CD patients. Vitamin D levels were inversely correlated with endoscopic disease activity. Vitamin D status could be a biomarker in assessing disease activity among hospitalized CD patients in addition to CDAI and CRP.

## 1. Introduction

Vitamin D (25OHD), as a potential immune modulator, is a risk factor for Crohn's disease (CD). Vitamin D influences both CD onset [1] and progression [2] of disease activity [3]. However, evidence regarding associations between vitamin D status and disease activity in CD has been inconsistent. A recent meta-analysis [4] showed CD patients have lower levels of vitamin D compared to healthy controls, but such difference disappeared when compared to nonhealthy controls [5-7]. Furthermore, vitamin D levels may correlate with disease activity among CD patients [8, 9].

Historically, clinical indices (Crohn's disease activity index (CDAI), Harvey-Bradshaw index (HBI)) and systemic inflammatory markers (C-reactive protein (CRP)) have been used to assess disease activity in CD and have been found to correlate with levels of vitamin D [10, 11]. However, compared to conventional clinical index and CRP in CD, endoscopic assessment of disease activity has been thought to be superior in discriminating disease severity and degrees of mucosal inflammation in clinical practice. For example, mucosal healing under endoscopy examination is strongly associated with favorable disease outcome and therefore it has become an increasingly important therapeutic goal for CD patients. Simple endoscopic score for Crohn's disease (SES-CD) is developed to assess mucosal inflammation in CD. SES-CD demonstrated high level of agreement among different endoscopists [12]. Furthermore, improvement of SES-CD scores in therapeutic trials was associated with corticosteroid-free clinical remission in patients with CD [13].

Vitamin D levels as an immune modulator may predict mucosal activity in patients in CD. To our knowledge, association between 25OHD and endoscopic disease severity assessed by SES-CD is unknown. Therefore, the primary aim of the current study is to investigate the association between vitamin D levels and endoscopic disease activity in CD using SES-CD. The secondary aim is to evaluate the association between vitamin D levels and other disease activity assessment tool such as CDAI, CRP, and albumin.

## 2. Material and Methods

### 2.1. Patient Population

All participants provided consents to participate in the current study. This study is approved by the ethics committee of our hospital. Consecutive CD patients hospitalized between March 2014 and July 2016 at the inflammatory bowel disease inpatient center of our hospital who received vitamin D level evaluation and colonoscopy were retrospectively analyzed. The 25OHD levels were compared in CD patients to 40 healthy controls and are comparable in terms of age, sex, and timing of the test. Inclusion criteria included (1) diagnosed as CD based on a careful evaluation on patients' history, imaging, lab tests, and endoscopic and pathology results, (2) hospitalized patients between March 2014 and July 2016 admitted to the inflammatory bowel disease inpatient center, (3) age ≥ 16 years, (4) those patients with colonic (L2) or ileocolonic (L3) disease according to Montreal classification. Patients who had previous extensive small intestinal surgery or short bowel syndrome were excluded given that vitamin D deficiency may be attributed to malabsorption. Clinical and endoscopic data were obtained to characterize the patient's clinical course.

### 2.2. Definitions of Crohn's Disease Activity

Crohn's disease activity was evaluated by a combination of clinical, biochemical, and endoscopic assessment. Crohn's disease activity index (CDAI) was calculated for each patient; disease severity was stratified to four categories according to CDAI scores, with CDAI< 150 classified as remission, CDAI between 150 and 220 as mildly active, CDAI between 220 and 450 as moderately active, and CDAI above 450 as severely active [14]. C-reactive protein (CRP) levels were obtained in all patients at presentation. Abnormal CRP was defined as a level of above 5 mg/L. Albumin is considered as a supplementary index for disease activity, and <35 g/L was considered abnormal. Colonoscopy was performed by one gastroenterologist, and endoscopic scores were graded by another experienced gastroenterologist specializing in IBD who reviewed all relevant stored images.

Disease activity assessed by endoscopy was calculated using the simple endoscopic score for Crohn's disease (SES-CD) (Table 1) [15]. Each of the four SES-CD variables (size of ulcers, ulcerated surface, affected surface, and the presence of narrowing) is scored from 0 to 3 for each of the five anatomical segments (terminal ileum, right colon, transverse colon, left colon, and rectum). The total SES-CD score is the sum of scores of each variable from the five locations, raging from 0 to 56, and is further categorized as no active disease (0–2), mildly active disease [3–6], moderately active disease [7–15], and severely active disease (>15) [15].

### 2.3. Definitions of Vitamin D Deficiency or Insufficiency

Serum 25OHD concentration was measured with radioimmunoassay and automeasured by using the Food and Drug Administration-approved Roche cobas 8000 automatic biochemical analyzer in the clinical laboratory of our hospital. According to the endocrine society clinical practice guideline [16], vitamin D deficiency is defined as a serum level of 25OHD lower than 50 nmol/L, and a serum level above 50 nmol/L but lower than 75 nmol/L is classified as vitamin D insufficiency.

### 2.4. Statistics

Continuous variables following normal or nonnormal distributions were interpreted as mean with standard deviations, and median with quartiles, respectively. Categorical variables were presented as proportions. Kruskal-Wallis test was used to compare nonparametric variables between groups. Mann–Whitney *U* test was used to compare bivariate variables. Kruskal-Wallis test and Spearman's rho were used to compare across categories of independent samples. Statistical significance was considered as a *P* value of lower than 0.05. Statistical analysis was performed with SPSS (version 22.0, Chicago, IL).

## 3. Results

### 3.1. Characteristics of Patients and Healthy Controls

A total of 131 patients with CD met the inclusion criteria. Forty healthy controls were subsequently recruited. The two groups were not statistically different in age, sex, timing of the assay, nonsmoking, and BMI (Table 2). Two patients were excluded due to history of extensive small bowel disease surgery. The characteristics of 131 patients are shown in Table 3. The median age was 27, 96 patients (73%) were males, and 120 (91.6%) were nonsmokers. The median time of disease duration was 2 years, and 105 patients (80.2%) were newly diagnosed with CD. Eight patients (6.1%) had colonic disease and 123 (93.9%) had ileocolonic disease. Of the 26 patients who was diagnosed before March 2014, 6 (23.1%) were being treated with corticosteroids, 12 (46.2%) received immunomodulators, 5 (19.2%) received mesalazine, and 3 (11.5%) received biologics at presentation.

### 3.2. Prevalence of Vitamin D Deficiency

The median serum 25OHD level of 131 patients was lower than healthy controls [21.1 nmol/L (11.8–32.3) versus 49.9 nmol/L (44.9–57.4), *P* = 0.007]. All patients had suboptimal levels of vitamin D including 125 (95%) with vitamin D deficiency and remaining 6 (5%) with vitamin D insufficiency.

### 3.3. Vitamin D Status and Disease Severity Measured by SES-CD

Endoscopic evaluation of disease severity showed that 8.4% of the patients was in clinical remission, 17.6% were mildly active, 43.5% had moderately active disease, and 30.5% had severely active disease. Disease severity, evaluated by SES-CD, was inversely correlated with 25OHD levels (*r* = −0.294, *P* = 0.001) (Table 4).

**Figure 1:**
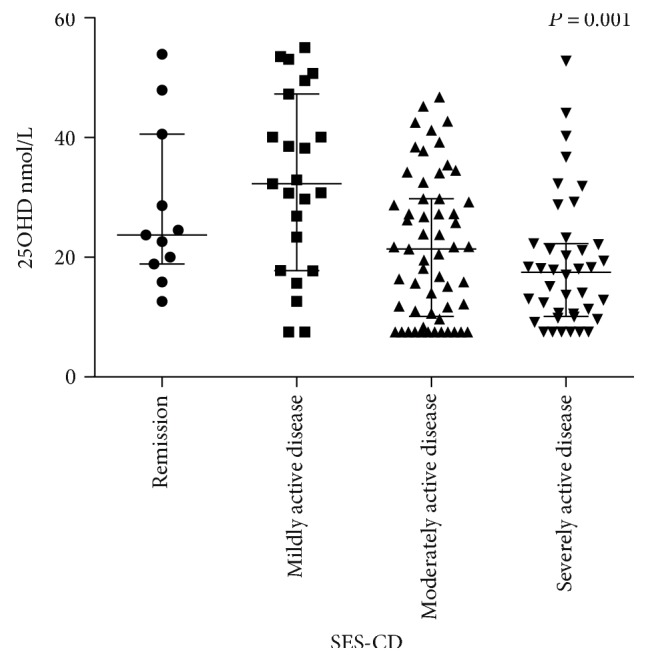
Comparison of serum 25OHD level of patients among clinical remission group, mildly active group, moderately active group, and severely active group stratified by SES-CD scores, *P* = 0.001.

Serum levels of 25OHD were lower in patients with active disease compared to patients in remission. For patients in remission, mild active disease, moderate active disease, and severe active disease, the serum 25OHD levels were 23.7 nmol/L, 32.3 nmol/L, 21.4 nmol/L, and 17.5 nmol/L, respectively (*P* = 0.001) (Figure 1).

### 3.4. Vitamin D Status and Disease Severity Measured by CRP, CDAI, and Albumin

The majority of subjects (82.4%) had a CRP level of higher than 5, indicating active inflammation, and the remaining 17.6% was in remission, indicated as a CRP level of lower than 5. At presentation, 115 (88%) patients were experiencing flare and 16 (12%) were in clinical remission, as indicated by CDAI. Of those with active disease, 32% was mildly active, 53.4% had moderately active disease, and 2.2% had severely active disease.

**Figure 2:**
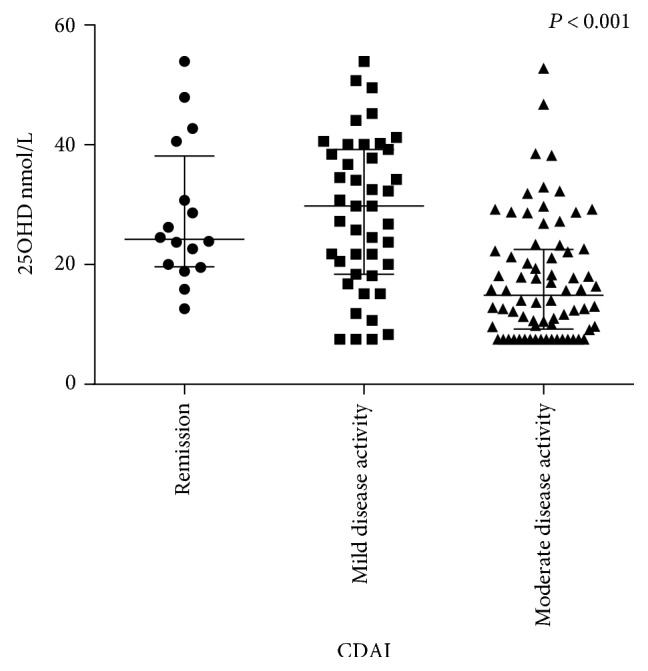
Comparison of serum 25OHD levels among clinical remission group, mildly active group, and moderately active group stratified by CDAI scores, *P* < 0.001.

Disease severity, evaluated by CRP, CDAI, and albumin, was inversely correlated with 25OHD levels (Table 4). For patients in clinical remission as defined by a CDAI score of lower than 150, the median serum level of 25OHD was 33.1 nmol/L, whereas in patients with mild disease and moderately active disease, the levels were lower (29.8 nmol/L and 14.8 nmol/L, respectively, *P* < 0.001) (Figure 2).

## 4. Discussion

Lower serum 25OHD concentration may be associated with greater disease activity [2, 17], poorer disease course, and worse outcomes in CD patients [18]. Many researches had been carried out to explore the relationships between vitamin D status, disease activity, and systemic markers of inflammation [2, 10, 17, 19, 20], and the results were inconsistent [3, 21]. Most studies have reported an inverse correlation between vitamin D status and disease activity in terms of HBI scores [11, 17, 19], CDAI, and CRP [17]. Yet, some other studies failed to demonstrate such associations between 25OHD with either CRP or CDAI [21–23]. Our study shows that disease severity, evaluated by systematic inflammation CRP, CDAI, and albumin, was strongly and inversely correlated with 25OHD levels, which is consistent with most studies.

To further investigate the association between vitamin D levels and clinical disease severity, we adopted SES-CD, in addition to CDAI and CRP levels; SES-CD was chosen to assess disease activity and mucosal inflammation. SES-CD is superior to CRP and CDAI in identifying various degrees of mucosal inflammation as it provides direct visualization of intestine mucosa. To our knowledge, there are no data that elucidate the relationship between vitamin D levels and endoscopic inflammation in patients with Crohn's disease. Our study is the first to report an inverse correlation between SES-CD and serum 25OHD concentrations, after analyzing the data from 131 patients with CD. Moreover, we found a significant, inverse association between serum 25OHD and four SES-CD categories, which indicated various degrees of disease severity.

Our study also finds that vitamin D deficiency is present in as many as 95.4% of inpatients with CD. A recent meta-analysis reported that the prevalence of vitamin D deficiency was 57.7% in CD patients [4]. A recent study shows that vitamin D deficiency accounts for 53% (122/230) in outpatients with CD [24]. The prevalence of vitamin D deficiency in our study was higher, probably because participants in our study were all inpatient and had more severe disease and because their disease activity has not been controlled since most of the participants were newly diagnosed.

The current study has its limitation. The sample size is not large enough due to its retrospective analysis in nature. The controversial role of vitamin D in the assessment of disease activity as well as response to treatment still needs evidence from further well-designed prospective study.

In conclusion, among hospitalized patients with CD who received a colonoscopy, almost all the patients had vitamin D deficiency. In addition to CDAI and CRP levels, vitamin D levels are highly correlated with endoscopic disease activity assessed by simple endoscopic score for Crohn's disease. Vitamin D status may be a useful biomarker in assessing disease activity among hospitalized patients with Crohn's disease in clinical practice.

## Figures and Tables

**Table 1:** Definitions of simple endoscopic score for Crohn's disease.

	Simple endoscopic score for Crohn's disease values			
	0	1	2	3
Size of ulcers	None	Aphthous ulcers (0.1–0.5 cm)	Large ulcers (0.5–2 cm)	Very large ulcers (>2 cm)
Ulcerated surface	None	<10%	10–30%	>30%
Affected surface	Unaffected segment	<50%	50–75%	>75%
Presence of narrowings	None	Single, can be passed	Multiple, can be passed	Cannot be passed

**Table 2:** Comparisons of characteristics between patient group and healthy controls.

Characteristics	Healthy controls *N* = 40 (%) patients with colorectal cancer	Patients with Crohn's disease *N* = 131 (%)	*P* value
Age [median (quartiles)]	27 (18–57)	27 (22–35)	0.646
Male	27 (67.5%)	96 (73%)	0.575
Serum 25OHD level, nmol/L [median (quartiles)]	49.9 (44.9–57.4)	21.1 (11.8–32.3)	0.007
Detected in spring and winter/Detected in summer and autumn	17/23	64/67	0.588
Nonsmoking [*n* (%)]	32 (80%)	120 (91.6%)	0.302
BMI [median (quartiles)]	20 (17.3–22.1)	19.0 (17.0–21.3)	0.701

BMI: body mass index.

**Table 3:** Clinical characteristics of patient group.

Characteristics	*N* = 131 (%)
Years diagnosed [median (quartiles)]	2 (1–5)
Montreal classification [*n* (%)]	
Age at diagnosis	
A1 (<17 years)	9 (6.8)
A2 (17–40 years)	99 (75.6)
A3 (≥40 years)	23 (17.6)
Disease location [*n* (%)]	
L2 (colonic)	8 (6.1)
L3 (ileocolonic)	123 (93.9)
Disease behavior [*n* (%)]	
B1 (nonstricturing, nonpenetrating)	97 (74)
B2 (stricturing)	24 (18.3)
B3 (penetrating)	4 (3.1)
B2 + B3(stricturing and penetrating)	6 (4.6)
Disease activity	
CDAI score [mean (SD)]	241 (84)
CRP, mg/L [median (quartiles)]	16.6 (6.4–37.1)
Albumin, g/L [mean (SD)]	34.8 (6.2)
SES-CD [median (quartiles)]	11 (6–17)
Serum 25OHD [nmol/L, median (quartiles)]	21.1 (11.8–32.3)

SES-CD: the simple endoscopic score for Crohn's disease, CDAI: Crohn's disease activity index, and CRP: C-reactive protein.

**Table 4:** Association between vitamin D status and disease severity in Crohn's disease, measured by CRP, CDAI, albumin, and SES-CD.

	Spearman's rho	*P* value
25OHD and SES-CD	−0.294	0.001
25OHD and CDAI	−0.582	<0.001
25OHD and CRP	−0.308	<0.001
25OHD and albumin	−0.484	<0.001

SES-CD: the simple endoscopic score for Crohn's disease, CDAI: Crohn's disease activity index, and CRP: C-reactive protein.
